# Supplementary material for: Sulfide catabolism ameliorates hypoxic brain injury
Source: Nat Commun. 2021 May 25;12:3108. doi: 10.1038/s41467-021-23363-x (PMC8149856; doi:10.1038/s41467-021-23363-x)
Supplement: Supplementary file 1 — Supplementary Information [file 41467_2021_23363_MOESM1_ESM.pdf]

Supplementary Information

Sulfide catabolism ameliorates hypoxic brain injury

Eizo Marutani[1,2,†], Masanobu Morita[3,†], Shuichi Hirai[1,2,†], Shinichi Kai[1,2], Robert M. H. Grange[1,2], Yusuke Miyazaki[1,2], Fumiaki Nagashima[1,2], Lisa Traeger[1,2], Aurora Magliocca[1,2], Tomoaki Ida[3], Tetsuro Matsunaga[3], Daniel R. Flicker[4,5,6], Benjamin Corman[1,7], Naohiro Mori[1,2], Yumiko Yamazaki[1], Annabelle Batten[1], Rebecca Li[1], Tomohiro Tanaka[8], Takamitsu Ikeda[1,2], Akito Nakagawa[1,2], Dmitriy N. Atochin[2,9], Hideshi Ihara[10], Benjamin A. Olenchock[2,11], Xinggui Shen[12], Motohiro Nishida[8,13], Kenjiro Hanaoka[14], Christopher G. Kevil[12], Ming Xian[15], Donald B. Bloch[1,2,7], Takaaki Akaike[3,‡], Allyson G. Hindle[1,2,16,‡], Hozumi Motohashi[17,‡,*], Fumito Ichinose[1,2,‡,*]

Affiliations:

1- Anesthesia Center for Critical Care Research of the Department of Anesthesia, Critical Care and Pain Medicine, Massachusetts General Hospital, Boston, MA
2- Harvard Medical School, Boston, MA
3- Department of Environmental Medicine and Molecular Toxicology, Tohoku University Graduate School of Medicine, Sendai 980-8575, Japan
4- Department of Systems Biology, Harvard Medical School, Boston, MA
5- Howard Hughes Medical Institute and Department of Molecular Biology, Massachusetts General Hospital, Boston, MA
6- Broad Institute of MIT and Harvard, Cambridge, MA
7- Division of Rheumatology, Allergy and Immunology, Department of Medicine, Massachusetts General Hospital, Boston, MA
8- Division of Cardiocirculatory Signaling, National Institute for Physiological Sciences & Exploratory Research Center on Life and Living Systems & Center for Novel Science Initiatives, National Institutes of Natural Sciences, Okazaki 444-8787, Japan
9- Cardiovascular Research Center, Division of Cardiology, Department of Medicine, Harvard Medical School, Massachusetts General Hospital, Charlestown, MA, USA
10- Department of Biological Science, Graduate School of Science, Osaka Prefecture University, Osaka, 599-8531, Japan
11- Division of Cardiovascular Medicine, Department of Medicine, The Brigham and Women's Hospital, Boston, MA
12- Department of Pathology and Translational Pathobiology, Louisiana State University Health Sciences Center-Shreveport, Shreveport, LA
13- Department of Physiology, Graduate School of Pharmaceutical Sciences, Kyushu University, Fukuoka 812-8582, Japan
14- Graduate School of Pharmaceutical Sciences, The University of Tokyo, 7-3-1 Hongo, Bunkyo-ku, Tokyo, Japan
15- Department of Chemistry, Brown University, RI
16- School of Life Sciences, University of Nevada Las Vegas, Las Vegas, NV
17- Department of Gene Expression Regulation, Institute of Development, Aging and Cancer, Tohoku University, Sendai 980-8575, Japan

†These authors contributed equally.
‡ These authors jointly supervised this work.
*Correspondence to H.M. (hozumim@med.tohoku.ac.jp) or F.I. (fichinose@mgh.harvard.edu)

Supplementary figures

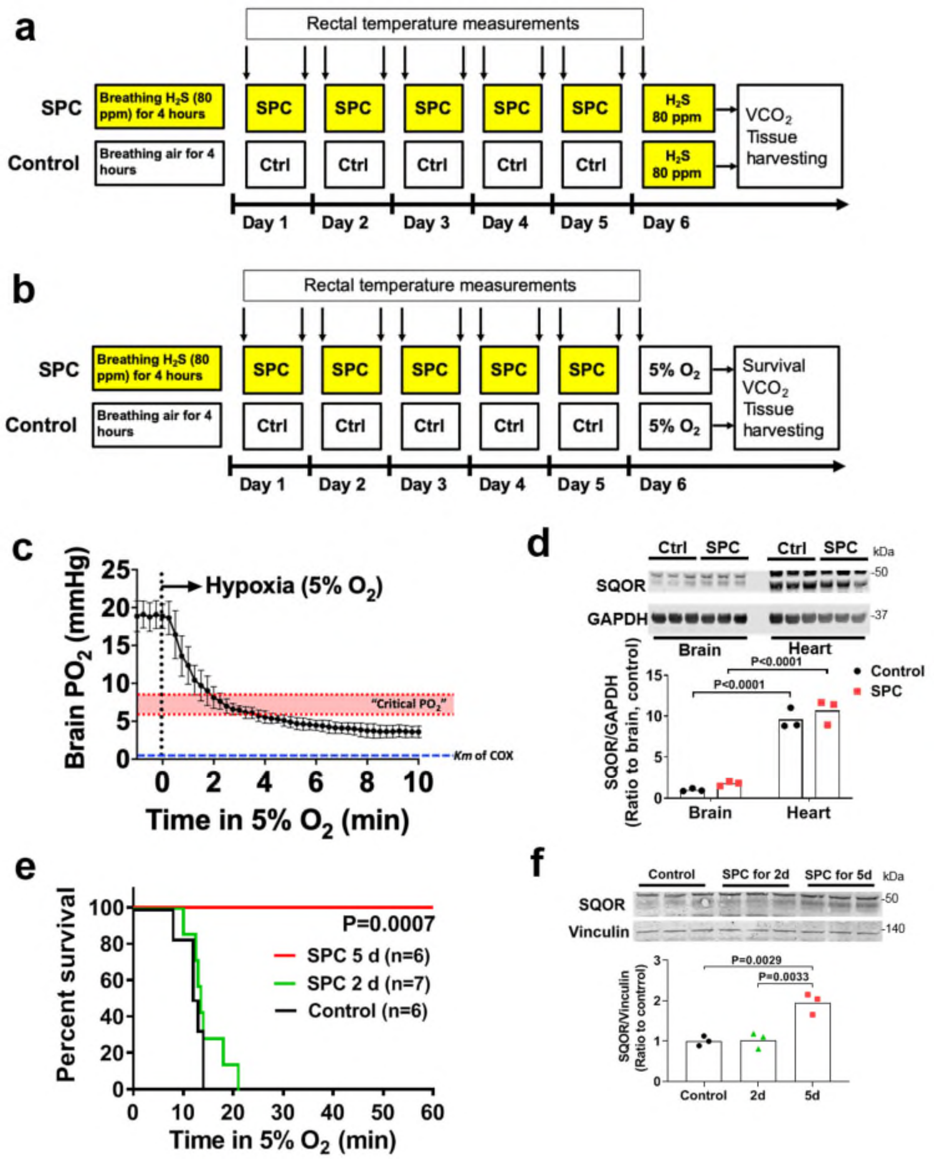

Supplementary Fig. 1: Effects of sulfide pre-conditioning. a Schematic representation of protocols of H_2S inhalation after sulfide preconditioning (SPC) and **b** hypoxia breathing after SPC. **c** Change of brain tissue PO_2 in mice ventilated with 5% oxygen under isoflurane anesthesia (n = 3). Data are shown as mean ± SD. Critical PO_2 = 6-8 mmHg at which brain tissue ATP levels start decreasing rapidly. Km of COX; Km value of cytochrome c oxidase for oxygen. **d** Levels of SQOR in the brain and heart of control or SPC mice (n = 3 each). **e** Survival curve of control mice and mice treated with SPC 2 days or SPC 5 days in 5% oxygen. **f** Brain SQOR levels in control mice and mice treated with SPC 2 days (2d, n = 3) or SPC 5 days (5d, n = 3). Data are presented as mean± SEM or mean and individual values. Two-way ANOVA followed by Sidak's correction or One-way ANOVA followed by Tukey's correction was performed for **d** and **f**, respectively. Survival rates were estimated using the Kaplan-Meier method and log-rank test was used to compare the survival curves between groups for panel **e**.

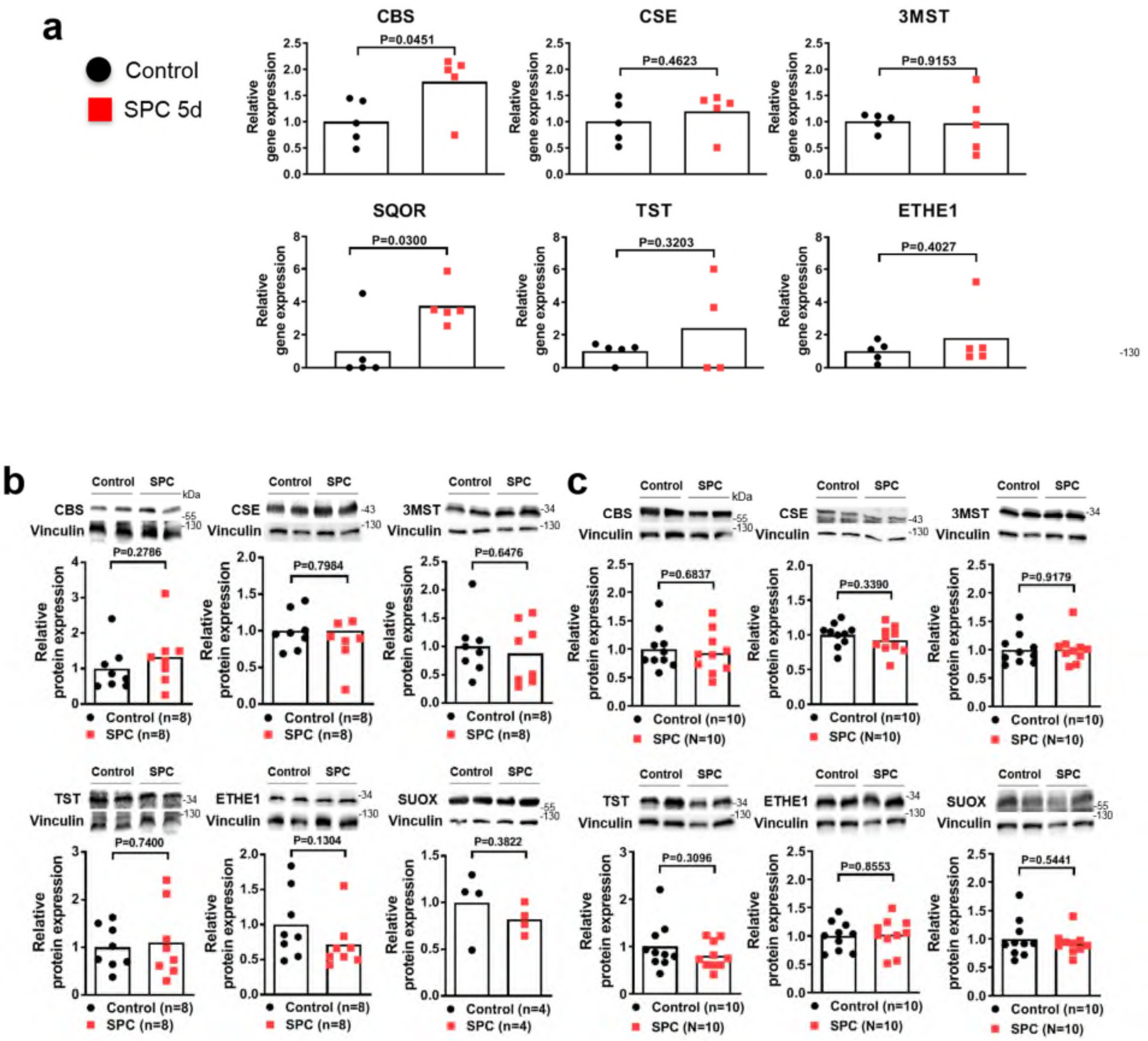

Supplementary Fig. 2: Effects of sulfide pre-conditioning on levels of enzymes that metabolize sulfide

a Relative mRNA levels of enzymes that synthesize or metabolize sulfide in brains in control mice and mice subjected to sulfide pre-conditioning (SPC). n = 5 each except TST. n = 5 and 4 for TST. CBS, cystathionine beta synthase. CSE, cystathionine gamma lyase. 3MST, 3-mercaptopyruvate sulfurtransferase TST, thiosulfate sulfurtransferase. ETHE1, ethylmaronic encephalopathy 1 (persulfide dioxygenase). **b** Representative immunoblot images and summary graphs of protein levels of enzymes in the brain that synthesize or metabolize sulfide in control mice and mice subjected to SPC. SUOX, sulfite oxidase. **c** Representative immunoblot images and summary graphs of protein levels of enzymes in the heart that synthesize or metabolize sulfide in control mice and mice subjected to sulfide SPC. Data are presented as mean and individual values. Two-tailed unpaired *t*-test was performed.

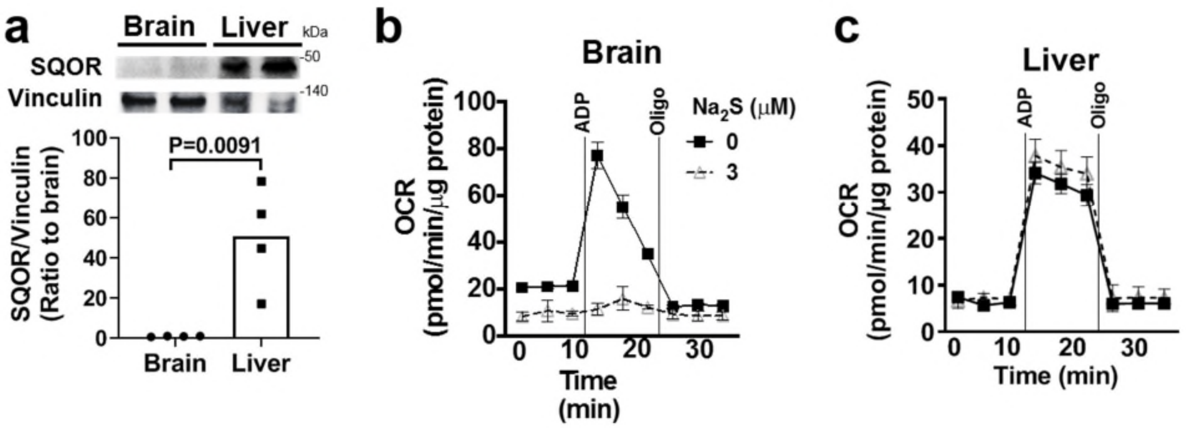

Supplementary Fig. 3: Comparison of SQOR levels between brain and liver

a Representative immunoblots and summary graph of the levels of SQOR in the brain and liver of naïve mice (n = 4 each). **b** Oxygen consumption rate (OCR) of isolated brain (n = 7 each) or **c** liver (n = 5 each) mitochondria from naïve mice with or without incubation with sulfide (Na_2S, 0, 3.0 µM). Data are presented as mean± SEM or mean and individual values. Two-tailed unpaired *t*-test was performed for **a**.

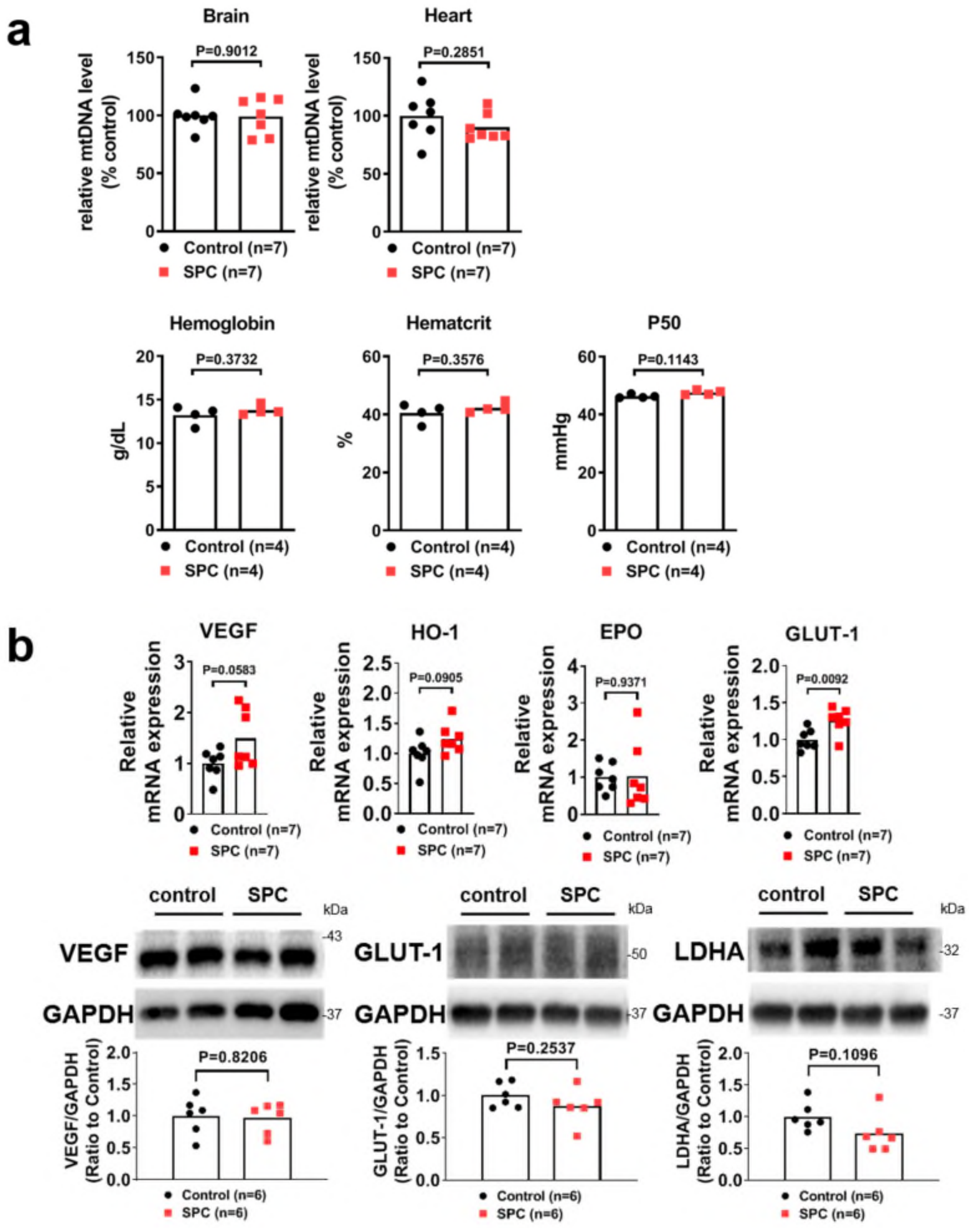

Supplementary Fig. 4: Other potential effects of sulfide pre-conditioning

a Effects of sulfide pre-conditioning (SPC) on mitochondrial DNA levels and red blood cells. Relative mitochondrial DNA levels in the brain and heart in control mice and mice subjected to SPC. Hemoglobin levels, hematocrit, and P50 of oxygen dissociation curve in control mice and mice subjected to SPC. **b** Effects of SPC on hypoxia inducible factor 1-dependent signaling. Relative gene expression levels of vascular endothelial growth factor (VEGF), homoxygenase-1 (HO-1), erythropoietin (EPO), glucose transporter 1 (GLUT1) and lactate dehydrogenase A (LDHA) in the brain in control mice and mice subjected to SPC. Protein levels of VEGF and GLUT1 in the brain in control mice and mice subjected to SPC. Data are presented as mean and individual values. Two-tailed unpaired *t*-test was performed.

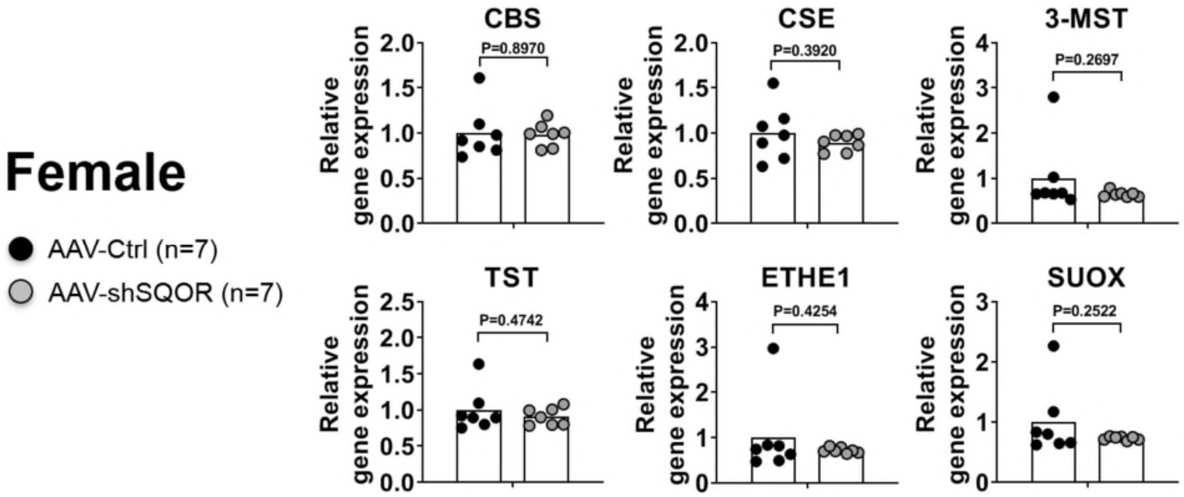

Supplementary Fig. 5: **Relative mRNA levels of enzymes that synthesize or metabolize sulfide in brains of adult female CD-1 mice infected with AAV-Ctrl or AAV-shSQOR.** CBS, cystathionine beta synthase. CSE, cystathionine gamma lyase. 3MST, 3-mercaptopyruvate sulfurtransferase. TST, thiosulfate sulfurtransferase. ETHE1, ethylmaronic encephalopathy 1 (persulfide dioxygenase). SUOX, sulfite oxidase. Data are presented as mean and individual values. Two-tailed unpaired *t*-test was performed.

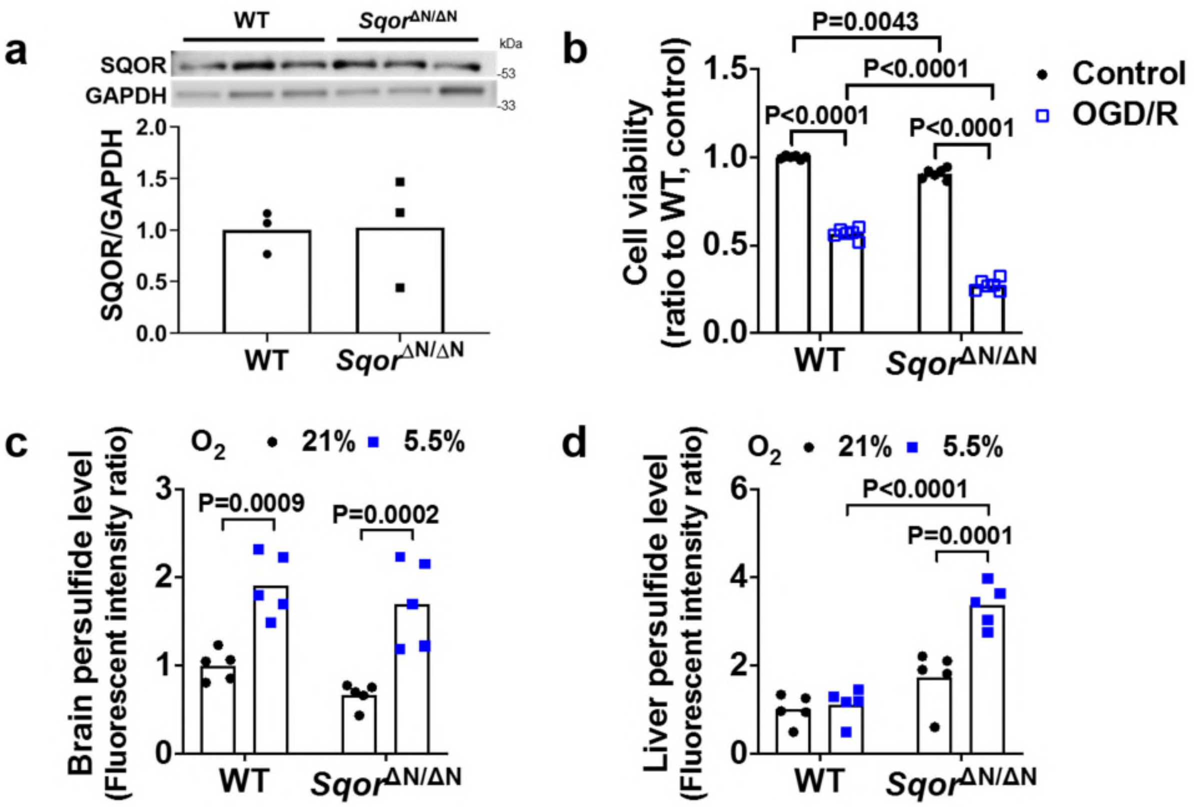

Supplementary Fig 6: **Effects of exclusion of SQOR from mitochondria. a** Immunoblots and densitometric quantification of SQOR levels in whole cell lysates of WT or *Sqor*$^{ΔN/ΔN}$ MEF cells using GAPDH as a loading control (n = 3 each). **b** Cell viability of primary cortical neurons obtained from WT or *Sqor*$^{ΔN/ΔN}$ mice subjected to oxygen glucose deprivation and reoxygenation (OGD/R) (*n* = 6 each). **c** Persulfide levels in brain (n = 5 each) and **d** liver (n = 5 each) of *Sqor*$^{ΔN/ΔN}$ mice and their wild type littermates breathing 21% or 5% oxygen. Data are presented as mean and individual values. Two-tailed unpaired *t*-test was performed for **a**. Two-way ANOVA followed by Sidak's correction was performed for panel **b**-**d**.

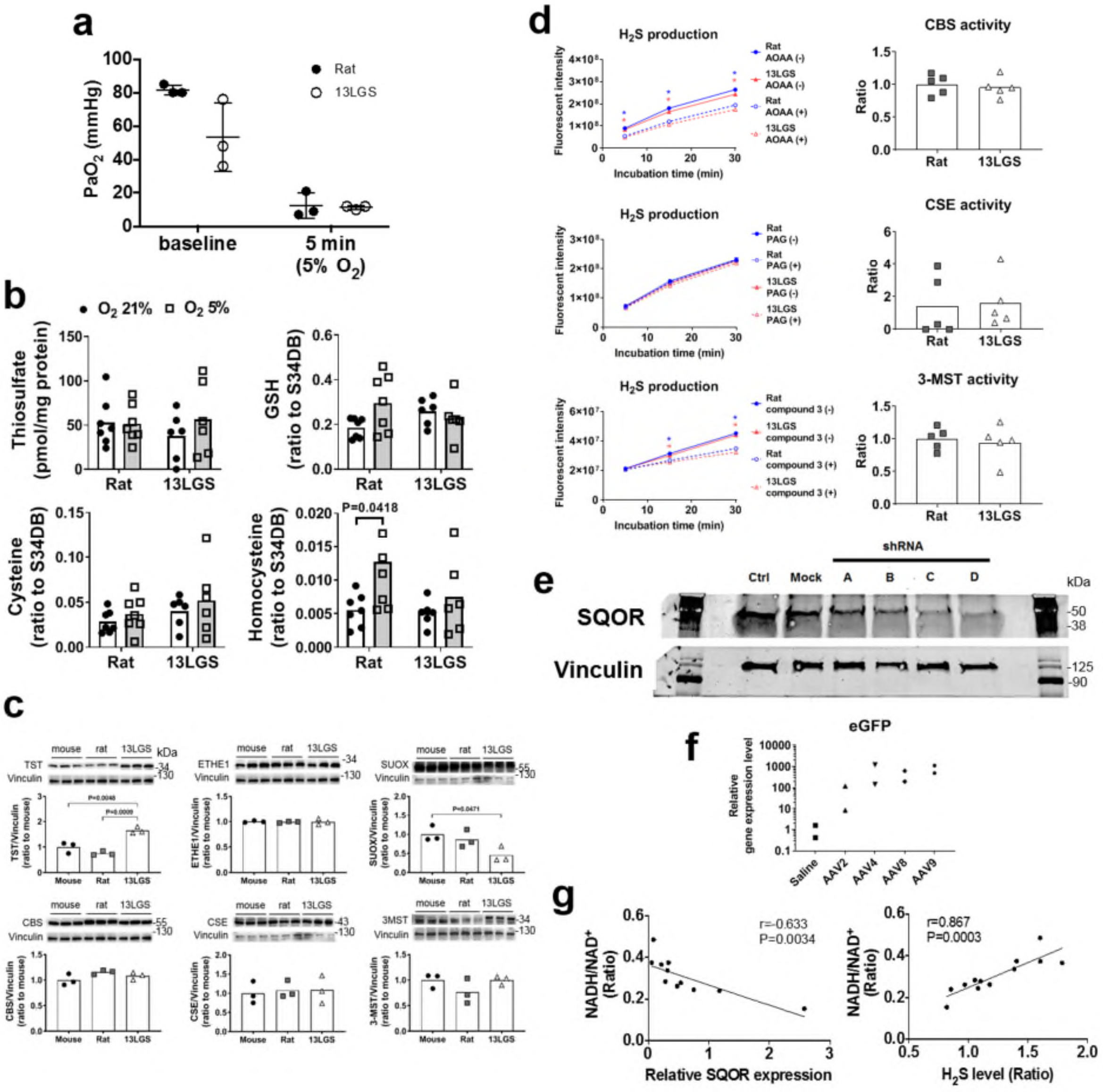

Supplementary Fig. 7: **13LG squirrel model**. **a** Changes in PaO_2 in rats and 13LG squirrels (13LGS) at baseline and after 5 min breathing 5% oxygen (n = 3 each). **b** Levels of thiosulfate, GSH, cysteine, and homocysteine in the brains of rats and 13LG squirrels after breathing 21% or 5% oxygen for 5 min (*n* = 7, 7, 6, 6). **c** Representative immunoblot images and summary graphs of protein expression levels of enzymes that synthesize or metabolize sulfide in brains of mouse, rat and 13LGS (n = 3 each). TST, thiosulfate sulfurtransferase. ETHE1, ethylmaronic encephalopathy 1 (persulfide dioxygenase). SUOX, sulfite oxidase. CBS, cystathionine beta synthase. CSE, cystathionine gamma lyase. 3MST, 3-mercaptopyruvate sulfurtransferase. **d** Enzyme activities of CBS, CSE, and 3MST in brains of rat and 13LGS (n = 5 each). AOAA, aminooxyacetic acid, CBS inhibitor; PAG, D,L-propargylglycine, CSE inhibitor; compound 3, 3MST inhibitor. **e** Knockdown effects of shRNA transfection on SQOR protein levels in primary myoblasts of 13LGS (representative image of 2 independent experiments). Protein levels of SQOR were measured at 48h after transfection by immunoblotting. **f** Brain GFP expression levels in 13LGS with ICV

injection of AAV. To determine the effective AAV serotype to induce gene transfer in 13LGS, AAV serotype 2, 4, 8, or 9 was injected ICV and brain GFP expression levels were compared using qPCR after 1 week (n = 2 each). **g** Correlation between gene expression level of SQOR and $NADH/NAD^+$ ratio and between relative H_2S level and $NADH/NAD^+$ ratio in the brain of 13LGS infected with AAV after breathing 5% oxygen for 5 min (n = 12). Data are presented as mean ± SEM or mean and individual values. Two-way ANOVA followed by Sidak's correction or one-way ANOVA followed by Tukey's correction was performed for **b** and **c**. Two-tailed unpaired *t*-test was performed for **d**. Two-tailed Pearson's coefficients were calculated for **g**.

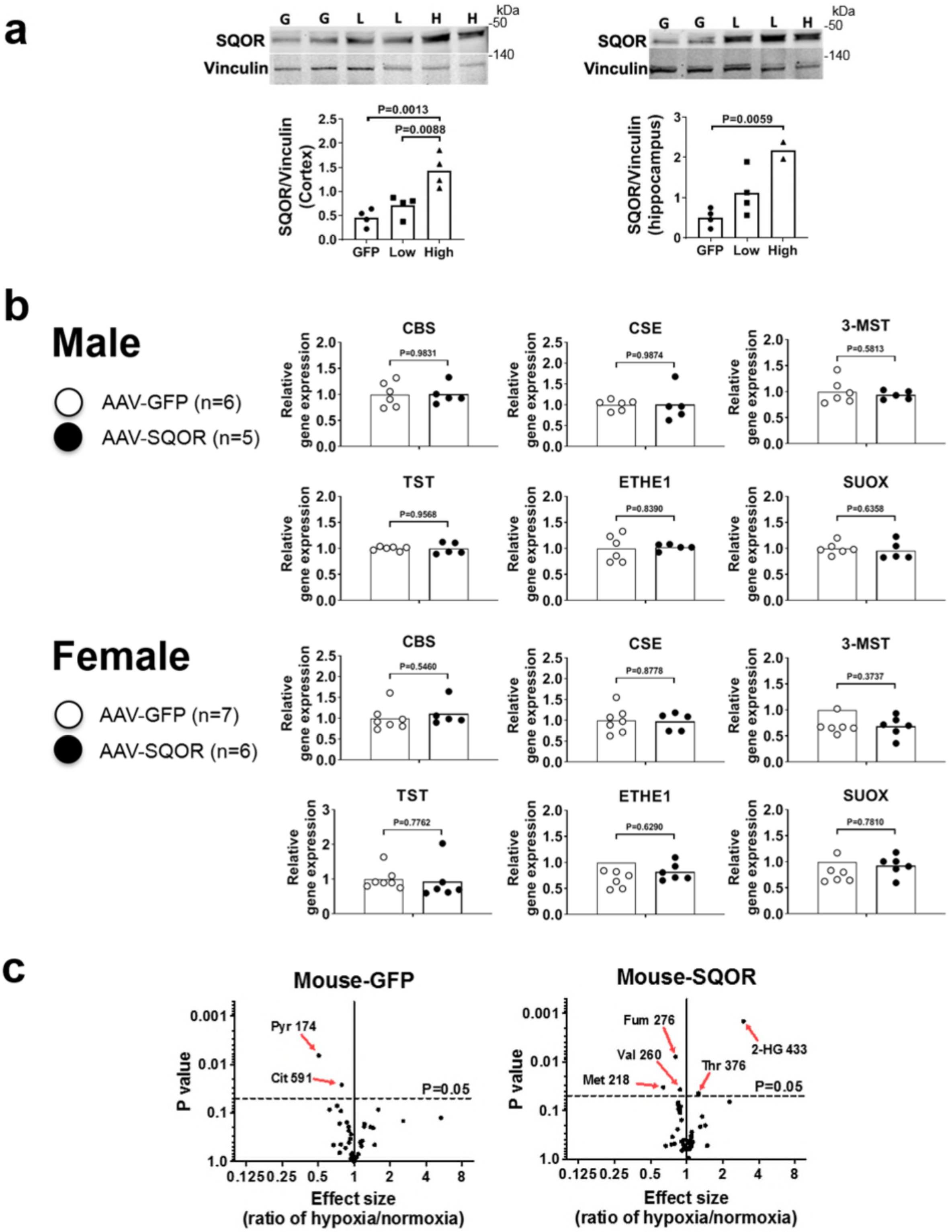

Supplementary Fig. 8: Effects of SQOR expression in the brain of mice. **a** SQOR protein levels in the cortex and hippocampus of the brains of mice that received AAV-GFP (G) or AAV-SQOR at

Low (L, 10^9) or High (H, 10^{10}) viral particles per hemisphere. **b** Relative gene expression levels of enzymes that synthesize or metabolize sulfide in brains of adult male and female CD-1 mice infected with AAV-GFP or AAV-SQOR. CBS, cystathionine beta synthase. CSE, cystathionine gamma lyase. 3MST, 3-mercaptopyruvate sulfurtransferase. TST, thiosulfate sulfurtransferase. ETHE1, ethylmaronic encephalopathy 1 (persulfide dioxygenase). SUOX, sulfite oxidase. **c** Volcano plots showing the changes in whole brain metabolite profiles in response to breathing 5.5 % oxygen in male CD-1 mice infected with AAV-GFP or AAV-SQOR. 2-HG, 2-hydroxyglutarate; Fum, fumarate; Lac, lactate; Met, methionine; Thr, threonine; Val, valine. Volcano plots were created using values in Table S4 and S5. One-way ANOVA followed by Tukey's correction was performed for **a**. Two-tailed unpaired *t*-test was performed for **b**.

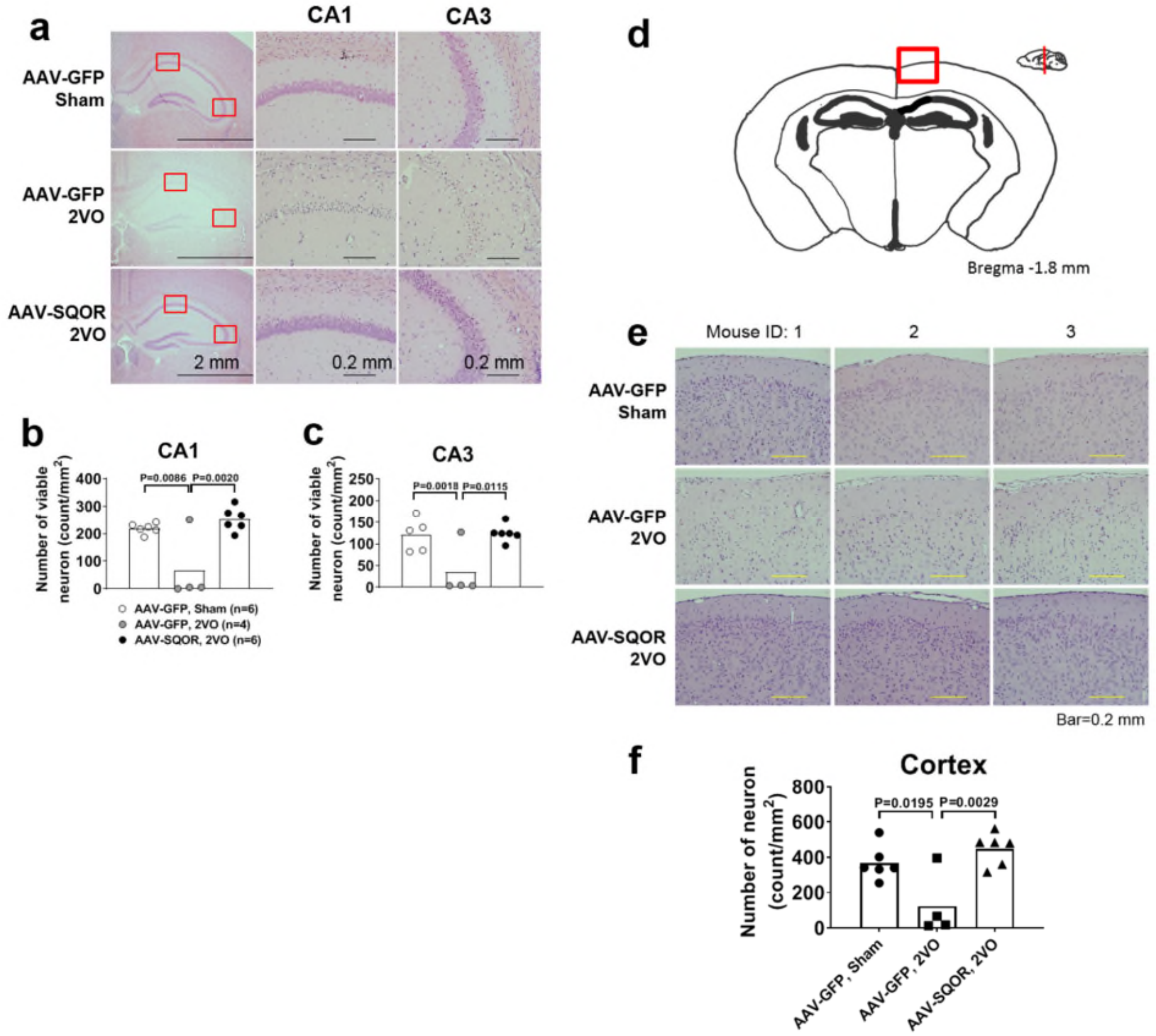

Supplementary Fig. 9: SQOR ameliorates ischemic brain injury in mice. A Representative photomicrographs of H&E-stained brain sections focusing on hippocampal CA1 and CA3 regions of male mice transfected with AAV-GFP and subjected to sham surgery (n = 6) or 2VO and reperfusion (n = 4) or AAV-SQOR and subjected to 2VO and reperfusion (n = 6). Number of viable neurons in **b** CA1 (n = 6, 4, 6.) and **c** CA3 (n = 5, 4, 6.) regions of mice transfected with AAV-GFP or AAV-SQOR and subjected to sham surgery or 2VO. **d** Mouse brain atlas showing the part of the brain cortex examined. **E** Representative photomicrograph of H&E-stained brain sections focusing on brain cortex of male mice transfected with AAV-GFP and subjected to sham surgery (n = 6) or 2VO and reperfusion (n = 4) or AAV-SQOR and subjected to 2VO and reperfusion (n = 6). **f** Summary of the number of viable neurons in brain cortex in mice subjected to sham operation or 2VO with or without SQOR expression (n = 6, 4, 6). Data are presented as mean and individual values. One-way ANOVA followed by Tukey's correction was performed for panel **a**, **c**, and **f**.

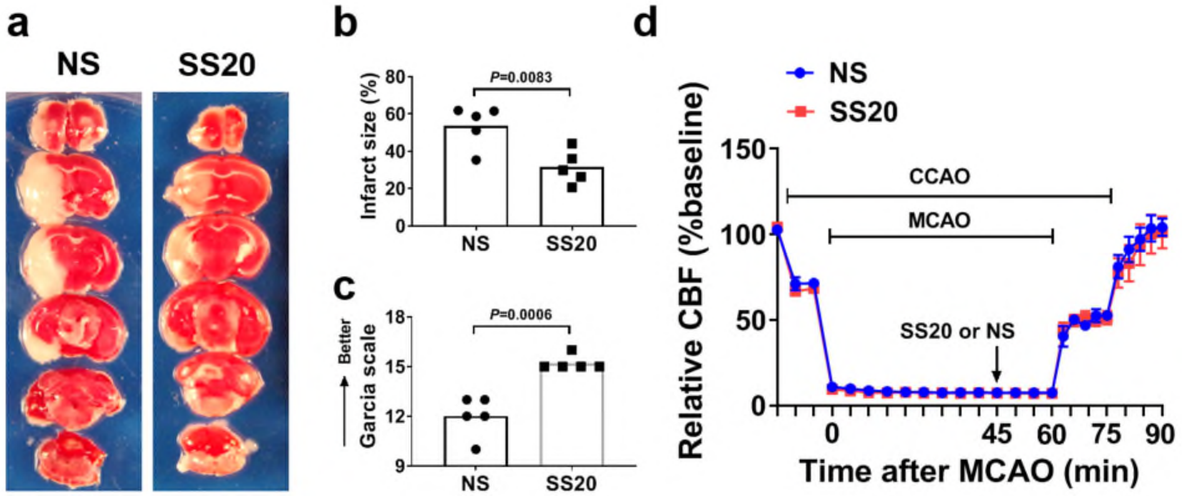

Supplementary Fig. 10: **Sulfide scavenger ameliorates ischemic brain injury in mice. a** Representative images of brain sections stained with TTC, **b** summary of infarct volume, and **c** neurological functional score after 60 min of transient MCAO and 48h of reperfusion in mice treated with normal saline or specific sulfide scavenger SS-20. **d** Relative cerebral blood flow in mice treated with normal saline or SS-20 15 min after the onset of ischemia. n = 5 each. Data are presented as mean and individual values or mean ± SEM. Two-tailed unpaired *t*-test was performed for **b** and **c**.

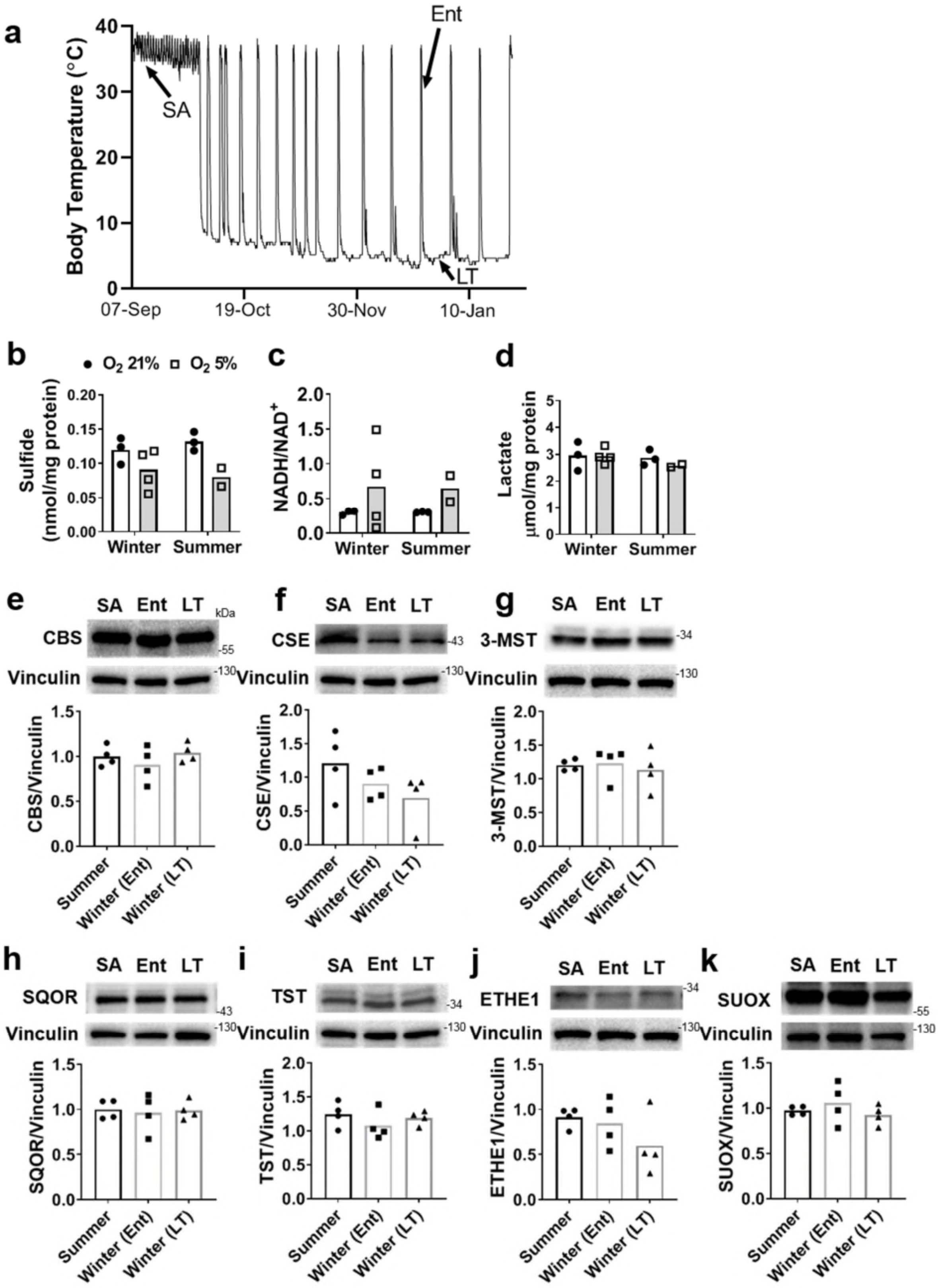
a
Body Temperature (°C)
Ent
SA
LT
07-Sep
19-Oct
30-Nov
10-Jan
b
O2 21%
O2 5%
Sulfide (nmol/mg protein)
Winter
Summer
c
NADH/NAD+
d
Lactate µmol/mg protein
e
SA
Ent
LT
kDa
CBS
Vinculin
CBS/Vinculin
Summer
Winter (Ent)
Winter (LT)
f
CSE
CSE/Vinculin
g
3-MST
3-MST/Vinculin
h
SQOR
SQOR/Vinculin
i
TST
TST/Vinculin
j
ETHE1
ETHE1/Vinculin
k
SUOX
SUOX/Vinculin

Supplementary Fig. 11: Sulfide metabolizing enzymes in summer and winter 13LG squirrels. a Annual body temperature trace of a 13LG squirrels in the laboratory, housed in a 4°C hibernaculum during the winter season. SA, summer active; Ent, Entrance to torpor bout; LT, late torpor during deep torpor. Brain levels of **b** sulfide, **c** NADH/NAD+ ratio, and **d** lactate of 13LGS breathed air or 5%O_2 for 5 min in winter or summer. n = 3, 4, 3, 2. **e-k** Representative immunoblot images and summary graphs of protein expression for enzymes in the brain that synthesize or metabolize sulfide in 13LG squirrels at three timepoints across the hibernation cycle. n = 4 each. Sampling timepoints are defined by body temperature and time of year depicted in **a**; summer active (SA) animals were sacrificed in August during the summer homeothermic period, Entrance (Ent) animals were collected during entrance into a torpor bout when body temperature was 27-23°C, late torpor (LT) animals were collected during deep torpor with body temperature near 4°C, typically after 7-10 days when the animal's bout reached 80-95% of the duration of its previous torpor bout. CBS, cystathionine beta synthase. CSE, cystathionine gamma lyase. 3MST, 3-mercaptopyruvate sulfurtransferase. SQOR, sulfide:quinone oxidoreductase. TST, thiosulfate sulfurtransferase. ETHE1, ethylmaronic encephalopathy 1 (persulfide dioxygenase). SUOX, sulfite oxidase. Data are presented as mean and individual values. Two-way ANOVA followed by Sidak's correction or one-way ANOVA with Tukey's correction was performed for panel **b**-**d** and **e**-**k**, respectively.

Supplementary tables

Supplementary Table 1. Mean ion counts normalized with AUC of brain metabolites measured by GC-MS in rats. n = 7 in each O_2 concentration. P-values were obtained by multiple *t*-test.

**Ion species**	**21% O_2**	**5% O_2**	**p-value**
Pyr_174	6.50849e-005	5.70086e-005	0.651026
Lac_223	0.00207663	0.00326733	0.013447
Lac_261	0.00243856	0.00505582	0.012296
Ala 232	0.000296135	0.000397022	0.124424
Ala 260	0.000253092	0.000332558	0.136735
Gly 218	0.000385092	0.000387856	0.957862
Gly 246	0.000325312	0.000323455	0.965953
Fum 287	0.000226795	0.000354567	0.007616
Ser 288	0.000570278	0.000590967	0.82665
Ser 302	0.000314642	0.000327459	0.806602
Ser 362	0.00051255	0.000538882	0.765693
Akg 346	5.85783e-006	4.42474e-006	0.356106
Mal 419	0.000273941	0.000322859	0.304859
Asp 302	0.00154742	0.00146013	0.661361
Asp 390	0.000617294	0.000589045	0.7384
Asp 418	0.00133539	0.00125778	0.669871
Glu 330	0.00413031	0.00330576	0.475835
Glu 432	0.0072204	0.00665123	0.414885
Gln 431	0.000738515	0.00119148	0.146789
Cit 459	0.000174346	0.000137175	0.156331
Cit 591	0.000116221	9.0486e-005	0.134994
Pro 330	3.71348e-005	3.73476e-005	0.954313
Pro 258	3.22401e-005	1.58194e-005	0.049187
Val 260	3.44283e-005	4.1471e-005	0.285143
Val 288	2.54186e-005	3.01869e-005	0.320469
Leu 200	0.000102373	0.000134716	0.095964
Leu 274	3.89868e-005	5.23065e-005	0.088079
Leu 302	3.31687e-005	4.38997e-005	0.096732
Leu 344	1.95873e-006	2.56482e-006	0.117771
Lie 200	4.1567e-005	5.60327e-005	0.099981
Lie 274	1.64376e-005	2.24511e-005	0.096407
Lie 302	2.01567e-005	2.71345e-005	0.095806
Lie 344	8.2475e-007	1.05877e-006	0.206482
Thr 376	2.63361e-005	3.56543e-005	0.190794
Thr 404	4.53716e-005	6.08905e-005	0.198932
Met 218	2.3905e-005	2.86905e-005	0.35611

Met 292	0.00210067	0.00133401	0.568965
Met 320	1.736e-005	2.06092e-005	0.306421
Phe 234	2.61922e-005	3.23615e-005	0.308761
Phe 302	3.56892e-005	4.48983e-005	0.251851
Phe 308	1.76954e-005	2.25202e-005	0.253396
Phe 336	2.90238e-005	2.80851e-005	0.55359
Cys 406	1.0156e-005	6.09951e-006	0.473979
Cys 378	0.00014205	0.000559118	0.032777
Tyr 302	0.000158229	0.0001915	0.422149
Tyr 364	1.63481e-005	2.06229e-005	0.341308
Tyr 466	2.28959e-005	2.8203e-005	0.379394
KMV 258	3.10446e-005	2.57922e-005	0.051912
KMV 216	8.5379e-007	7.39375e-007	0.471514
KIC 258	3.4541e-005	3.05453e-005	0.176268
KIC 216	1.113e-006	3.80472e-007	0.246484
2-HG 433	1.8076e-005	3.05519e-005	0.066508
Suc 289	0.00018282	0.000270558	0.032552

Supplementary Table 2. Mean ion counts normalized with AUC of brain metabolites measured by GC-MS in 13LG squirrels. n = 4 and 6 in 21% and 5% O_2, respectively. P-values were obtained by multiple *t*-test.

**Ion species**	**21% O_2**	**5% O_2**	**p-value**
Pyr_174	8.09277e-005	6.36684e-005	0.545939
Lac_223	0.00197282	0.00285291	0.154553
Lac_261	0.00309138	0.00444642	0.157632
Ala 232	0.000296238	0.000378651	0.123836
Ala 260	0.000252617	0.000317967	0.121951
Gly 218	0.000284217	0.000326946	0.290805
Gly 246	0.000239093	0.000272902	0.310442
Fum 287	0.000331525	0.000369185	0.10473
Ser 288	0.000499248	0.000467855	0.732694
Ser 302	0.00027458	0.000257181	0.731903
Ser 362	0.000450396	0.000423199	0.752393
Akg 346	9.86086e-006	9.10528e-006	0.838412
Mal 419	0.000352147	0.000413016	0.332212
Asp 302	0.00105998	0.000783834	0.146989
Asp 390	0.000425112	0.000312943	0.159099
Asp 418	0.000907128	0.0006691	0.162115
Glu 330	0.00424721	0.00427514	0.936842
Glu 432	0.00671319	0.00676504	0.923456
Gln 431	0.00114892	0.00115654	0.98526
Cit 459	0.000276084	0.000226374	0.405498
Cit 591	0.000183184	0.000150265	0.401444
Pro 330	4.60997e-005	5.15606e-005	0.296345
Pro 258	1.79219e-005	1.67094e-005	0.468645
Val 260	3.60448e-005	4.34371e-005	0.239119
Val 288	2.63329e-005	3.1435e-005	0.254951
Leu 200	8.16983e-005	0.00010981	0.132769
Leu 274	3.17085e-005	4.25038e-005	0.151474
Leu 302	2.68247e-005	3.54497e-005	0.156856
Leu 344	1.48254e-006	2.06859e-006	0.145074
Lie 200	3.75479e-005	5.137e-005	0.091458
Lie 274	1.51079e-005	2.06873e-005	0.09846
Lie 302	1.83958e-005	2.50198e-005	0.088474
Lie 344	6.94705e-007	1.0034e-006	0.10693
Thr 376	2.4128e-005	2.82607e-005	0.551927
Thr 404	4.10756e-005	4.78213e-005	0.566799
Met 218	2.42847e-005	2.15023e-005	0.62984
Met 292	0.00119967	0.00221315	0.625278
Met 320	3.36892e-005	1.6461e-005	0.142129

Phe 234	2.07365e-005	2.25621e-005	0.686096
Phe 302	2.86321e-005	3.13527e-005	0.662265
Phe 308	1.44024e-005	1.5513e-005	0.716458
Phe 336	2.07673e-005	2.00113e-005	0.830452
Cys 406	1.30451e-005	1.82628e-005	0.555365
Cys 378	0.000802204	0.000968002	0.671621
Tyr 302	0.000157799	0.000162267	0.906968
Tyr 364	1.7974e-005	1.81237e-005	0.974549
Tyr 466	2.33985e-005	2.8455e-005	0.361553
KMV 258	2.53622e-005	2.42613e-005	0.730993
KMV 216	4.9112e-007	6.33941e-007	0.350748
KIC 258	1.5794e-005	2.51876e-005	0.34791
KIC 216	1.2875e-006	3.79712e-007	0.088175
2-HG 433	6.77557e-005	8.63824e-005	0.356986
Suc 289	0.000176539	0.000278869	0.013109

Supplementary Table 3. Target sequences of shRNA for SQOR knockdown in 13LGS.

shRNA ID	Target sequence
**A**	5'-ATCTTTACCTTCCCAAATACTCC-3'
**B**	5'-AGCCTTTCTTCGGAAATTGTTTC-3'
**C**	5'-GACTGGCTACAACCGTGTGAT-3'
**D**	5'-GGCTACAACCGTGTGATTCTT-3'

Supplementary Table 4. Mean ion counts normalized with AUC of brain metabolites measured by GC-MS in mice with AAV-GFP injection. n = 6 in each O_2 concentration. P-values were obtained by multiple *t*-test.

**Ion species**	**21% O_2**	**5.5% O_2**	**p-value**
Pyr_174	0.000539	0.000272	0.006477
Lac_223	0.003137	0.003823	0.167089
Lac_261	0.004795	0.005805	0.188472
Ala 232	0.000238	0.00027	0.412577
Ala 260	0.000194	0.001031	0.125548
Gly 218	0.00025	0.00025	0.988683
Gly 246	0.000205	0.000158	0.441068
Suc 289	0.000576	0.000675	0.374188
Fum 287	0.000736	0.00057	0.090516
Ser 288	0.000402	0.000406	0.932201
Ser 302	0.000218	0.000217	0.991986
Ser 362	0.000347	0.000351	0.933217
Akg 346	5.39E-06	4.07E-06	0.656861
Mal 419	0.000274	0.000266	0.793683
Asp 302	0.00108	0.001118	0.839385
Asp 390	0.00042	0.00044	0.79014
Asp 418	0.000875	0.000912	0.811702
Glu 330	0.004011	0.003874	0.667459
Glu 432	0.006462	0.006238	0.666911
Gln 431	0.001118	0.001096	0.901008
Cit 459	0.000301	0.000216	0.070415
Cit 591	0.000123	9.73E-05	0.026144
Val 260	1.81E-05	2.68E-05	0.448056
Val 288	1.49E-05	1.39E-05	0.338841
Leu 200	0.000155	0.00014	0.192359
Leu 274	3.82E-05	3.59E-05	0.86643
Leu 302	4.69E-05	3.54E-05	0.164008
Leu 344	2.71E-06	2.45E-06	0.227199
ILe 200	0.000106	6.57E-05	0.085068
ILe 274	6.19E-06	5.25E-06	0.518119
ILe 302	3.08E-05	2.9E-05	0.272191
ILe 344	1E-06	1.16E-06	0.480405
Thr 376	1.36E-05	1.29E-05	0.687262
Thr 404	2.32E-05	2.23E-05	0.745873
Met 218	4.97E-05	6.79E-05	0.194199
Met 292	0.003677	0.004161	0.392086
Phe 234	6.56E-05	6.42E-05	0.929769
Phe 302	6.76E-05	6.13E-05	0.406002
Phe 308	0.000159	0.000138	0.154709
Cys 406	3.62E-05	5.21E-05	0.20179

Cys 378	0.000115	0.000297	0.14572
Tyr 302	0.000248	0.000235	0.722719
Tyr 364	4.19E-05	4.54E-05	0.700411
Tyr 466	3.31E-05	5.27E-05	0.084794
2-HG 433	0.000121	8.08E-05	0.4538

Supplementary Table 5. Mean ion counts normalized with AUC of brain metabolites measured by GC-MS in mice with AAV-SQOR injection. n = 6 in each O_2 concentration. P-values were obtained by multiple *t*-test.

**Ion species**	**21% O_2**	**5.5% O_2**	**p-value**
Pyr_174	0.000484	0.000437	0.435505
Lac_223	0.002829	0.002998	0.549406
Lac_261	0.004294	0.0045	0.631379
Ala 232	0.000235	0.00025	0.588639
Ala 260	0.000192	0.000203	0.616593
Gly 218	0.000279	0.000234	0.242928
Gly 246	0.000202	0.00016	0.481747
Suc 289	0.000539	0.000507	0.623679
Fum 287	0.000796	0.000642	0.007768
Ser 288	0.000365	0.000376	0.602888
Ser 302	0.000196	0.000205	0.462786
Ser 362	0.000315	0.000332	0.416795
Akg 346	7.77E-06	5.18E-06	0.533199
Mal 419	0.000286	0.000277	0.626874
Asp 302	0.001028	0.000871	0.067829
Asp 390	0.000405	0.000345	0.092723
Asp 418	0.000843	0.000712	0.074042
Glu 330	0.003848	0.00337	0.095731
Glu 432	0.006185	0.005424	0.110822
Gln 431	0.001265	0.001192	0.503399
Cit 459	0.000351	0.000319	0.669537
Cit 591	0.000134	0.000127	0.401638
Val 260	2.12E-05	1.87E-05	0.036663
Val 288	1.5E-05	1.34E-05	0.082206
Leu 200	0.000152	0.000164	0.409117
Leu 274	4.51E-05	4.65E-05	0.928291
Leu 302	4.65E-05	5.04E-05	0.365929
Leu 344	2.65E-06	2.89E-06	0.329923
ILe 200	6.81E-05	7.36E-05	0.334069
ILe 274	6.44E-06	9.52E-06	0.52565
ILe 302	2.99E-05	3.3E-05	0.278047
ILe 344	1.22E-06	9.3E-07	0.348401
Thr 376	1.43E-05	1.8E-05	0.044041
Thr 404	2.49E-05	3.1E-05	0.045649

Met 218	6.19E-05	3.94E-05	0.033052
Met 292	0.002331	0.003339	0.201567
Phe 234	6.11E-05	8.22E-05	0.133413
Phe 302	6.81E-05	7.25E-05	0.500808
Phe 308	0.000167	0.000151	0.155933
Cys 406	2.02E-05	4.61E-05	0.065585
Cys 378	0.000148	0.000111	0.496797
Tyr 302	0.000258	0.000283	0.538845
Tyr 364	3.72E-05	4.84E-05	0.21447
Tyr 466	3.46E-05	3.88E-05	0.440309
2-HG 433	6.68E-05	0.000199	0.00145

Supplementary Table 6. Breakdown of number of rats and 13LG squirrels used in each experiment.

Fig. 4b, sulfide level

Normoxia		***Hypoxia***		***Normoxia***				***Hypoxia***			
**Rat**		**Rat**		**13LGS (winter)**		**13LGS (summer)**		**13LGS (winter)**		**13LGS (summer)**	
**Male**	**Female**	**Male**	**Female**	**Male**	**Female**	**Male**	**Female**	**Male**	**Female**	**Male**	**Female**
3	4	3	4	1	2	2	1	2	2	1	1

Fig. 4c and d, lactate and NADH/NAD^+

Normoxia		***Hypoxia***		***Normoxia***				***Hypoxia***			
**Rat**		**Rat**		**13LGS (winter)**		**13LGS (summer)**		**13LGS (winter)**		**13LGS (summer)**	
**Male**	**Female**	**Male**	**Female**	**Male**	**Female**	**Male**	**Female**	**Male**	**Female**	**Male**	**Female**
3	3	3	3	1	2	2	1	2	2	1	1

Fig. 4e and f, volcano plot

Normoxia		***Hypoxia***		***Normoxia***				***Hypoxia***			
**Rat**		**Rat**		**13LGS (winter)**		**13LGS (summer)**		**13LGS (winter)**		**13LGS (summer)**	
**Male**	**Female**	**Male**	**Female**	**Male**	**Female**	**Male**	**Female**	**Male**	**Female**	**Male**	**Female**
3	4	3	4	1	2	1	0	2	2	1	1

Fig. 4g and Supplementary Fig. 11, protein expression

**Rat**		**13LGS (winter)**		**13LGS (summer)**	
**Male**	**Female**	**Male**	**Female**	**Male**	**Female**
1	2	1	2	0	0

Fig. 4h and Supplementary Fig. 12, enzymatic activity

**Enzyme**	**Rat**		**13LGS (winter)**		**13LGS (summer)**	
	**Male**	**Female**	**Male**	**Female**	**Male**	**Female**
**SQOR**	3	3	1	2	1	2
**CBS**	3	2	1	2	1	1
**CSE**	3	2	1	2	1	1
**3-MST**	3	2	1	2	1	1

Fig. 4, i and j, mitochondrial OCR

Rat		13LGS (winter)		13LGS (summer)	
Male	Female	Male	Female	Male	Female
1	5	0	0	2	4

Fig. 4, l-o, SQOR expression, sulfide, persulfide and NADH/NAD^{+}

13LGS (summer)			
Control		SQOR KD	
Male	Female	Male	Female
4	2	3	3

Supplementary Table 7: List of primer sequences for real-time PCR in mice

18S<br>18S	Forward<br>Reverse	5’-CGGCTACCACATCCAAGGAA-3’<br>5’-GCTGGAATTACCGCGGCT-3’
SQOR<br>SQOR	Forward<br>Reverse	5’-TGGGGACCTTCAGGATCTAA-3’<br>5’-GGACTGGAGACAACAGTGACC-3’
TST<br>TST	Forward<br>Reverse	5’-CCAGCTGGTGGACTCTCG-3’<br>5’-GTGGCCCGAGTCTAGTCCT-3’
ETHE1<br>ETHE1	Forward<br>Reverse	5’-CTGTCATCTCCCGCCTCA-3’<br>5’-GCTCGAGTCTCCAAAGCAA-3’
SUOX<br>SUOX	Forward<br>Reverse	5’-TCTACCATGAGCATCGGTGT-3’<br>5’-CATCGAAGACCTCAGAGCCTA-3’
CBS<br>CBS	Forward<br>Reverse	5'-CGGACTCCCCACATTATCAC-3‘<br>5'-CACACTTGAGACCGGCATTC-3’
CSE<br>CSE	Forward<br>Reverse	5'-CTTGCTGCCACCATTACGATT-3’<br>5'-TCTTCAGTCCAAATTCAGATGCC-3’
3MST<br>3MST	Forward<br>Reverse	5'-TCACAGCCGCTGAAGTTACTG-3’<br>5'-CAGCATGTGGTCGTAGGGG-3'
VEGF<br>VEGF	Forward<br>Reverse	5’-CCACGTCAGAGAGCAACATCA-3’<br>5’-TCATCTCTCCTATGTGCTGGCTTT-3’
HO-1<br>HO-1	Forward<br>Reverse	5’-CTGACCCATGACACCAAGGAC-3’<br>5’-AAAGCCCTACAGCAACTGTCG-3’
EPO<br>EPO	Forward<br>Reverse	5’-CATCTGCGACAGTCGAGTTCTG-3’<br>5’-CACAACCCATCGTGACATTTTC-3’
GLUT-1<br>GLUT-1	Forward<br>Reverse	5’-AGCCCTGCTACAGTGTATCCT-3’<br>5’-CCGACCCTCTTCTTTCATCT-3’
COX1<br>COX1	Forward<br>Reverse	5’-TGCTAGCCGCAGGCATTAC-3’<br>5’-GGGTGCCCAAAGAATCAGAAC-3’
NDUFV1<br>NDUFV1	Forward<br>Reverse	5’-CTTCCCCACTGGCCTCAAG-3’<br>5’-CCAAAACCCAGTGATCCAGC-3’

Supplementary Table 8

The optimum collision energy and precursor/production ions for thiol derivetives

	Precursor Ion (m/z)	Production Ion (m/z)	Dwells (s)	Collision (v)
$S^{34}DB$	417.4	192.1	0.005	30
Cysteine	312.3	192.1	0.005	25
HomoCys	326.3	192.1	0.005	30
Glutathione	498.5	192.1	0.005	35
