# Supplementary material for: Sulfide catabolism ameliorates hypoxic brain injury
Source: Nat Commun. 2021 May 25;12:3108. doi: 10.1038/s41467-021-23363-x (PMC8149856; doi:10.1038/s41467-021-23363-x)
Supplement: Supplementary file 3 — Reporting summary [file 41467_2021_23363_MOESM3_ESM.pdf]

nature research

Corresponding author(s): Eizo Marutani and Fumito Ichinose

Last updated by author(s): Mar 25, 2021

Reporting Summary

Nature Research wishes to improve the reproducibility of the work that we publish. This form provides structure for consistency and transparency in reporting. For further information on Nature Research policies, see our Editorial Policies and the Editorial Policy Checklist.

Please do not complete any field with "not applicable" or n/a. Refer to the help text for what text to use if an item is not relevant to your study.
For final submission: please carefully check your responses for accuracy; you will not be able to make changes later.

Statistics

For all statistical analyses, confirm that the following items are present in the figure legend, table legend, main text, or Methods section.

n/a	Confirmed	
☐	☒	The exact sample size (*n*) for each experimental group/condition, given as a discrete number and unit of measurement
☐	☒	A statement on whether measurements were taken from distinct samples or whether the same sample was measured repeatedly
☐	☒	The statistical test(s) used AND whether they are one- or two-sided<br>*Only common tests should be described solely by name; describe more complex techniques in the Methods section.*
☒	☐	A description of all covariates tested
☐	☒	A description of any assumptions or corrections, such as tests of normality and adjustment for multiple comparisons
☐	☒	A full description of the statistical parameters including central tendency (e.g. means) or other basic estimates (e.g. regression coefficient) AND variation (e.g. standard deviation) or associated estimates of uncertainty (e.g. confidence intervals)
☐	☒	For null hypothesis testing, the test statistic (e.g. F, t, r) with confidence intervals, effect sizes, degrees of freedom and P value noted<br>*Give P values as exact values whenever suitable.*
☒	☐	For Bayesian analysis, information on the choice of priors and Markov chain Monte Carlo settings
☒	☐	For hierarchical and complex designs, identification of the appropriate level for tests and full reporting of outcomes
☐	☒	Estimates of effect sizes (e.g. Cohen's *d*, Pearson's *r*), indicating how they were calculated

Our web collection on statistics for biologists contains articles on many of the points above.

Software and code

Policy information about availability of computer code

Data collection	Data collection and transformation was performed with the MassLynxTM 4.1 software in the MS-based measurements of sulfide metabolites.
Data analysis	Statistical analysis was performed using GraphPad Prism 8.4.3 (GraphPad Software, La Jolla, CA).

For manuscripts utilizing custom algorithms or software that are central to the research but not yet described in published literature, software must be made available to editors and reviewers. We strongly encourage code deposition in a community repository (e.g. GitHub). See the Nature Research guidelines for submitting code & software for further information.

Data

Policy information about availability of data

All manuscripts must include a data availability statement. This statement should provide the following information, where applicable:

- Accession codes, unique identifiers, or web links for publicly available datasets
- A list of figures that have associated raw data
- A description of any restrictions on data availability

The data that support the findings of this study are available from the corresponding author upon request.

Field-specific reporting

Please select the one below that is the best fit for your research. If you are not sure, read the appropriate sections before making your selection.

☒ Life sciences ☐ Behavioural & social sciences ☐ Ecological, evolutionary & environmental sciences

For a reference copy of the document with all sections, see nature.com/documents/nr-reporting-summary-flat.pdf

Life sciences study design

All studies must disclose on these points even when the disclosure is negative.

Sample size	A minimum sample size of 5 was used per group for in the majority of in vivo and in vitro experiments, as is standard and comparable to prior published experiments using similar techniques. The sample size is sufficient to detect a difference between two groups with coefficient of variation of 0.5 (ratio of standard deviation to mean difference) and two tailed level of significance (alpha) of 0.05 with a power of 80%. A smaller sample size of 3 was used in a limited number of experiments when the availability of sample is scarce and or the difference is expected to be large with small variation (e.g., brain samples of 13LG squirrels and brain SQOR levels in control mice). In these cases, the sample size is sufficient to detect a difference between two groups with coefficient of variation of 0.8 (ratio of standard deviation to mean difference) and two tailed level of significance (alpha) of 0.05 with a power of 80%.
Data exclusions	No data was excluded.
Replication	All experiments were performed at least twice and results were reliably reproduced. Independent biologic replicates are shown in all figures.
Randomization	We used randomized paired (a.k.a. matched pairs) design. We paired animals to two or more treatment groups on the basis of similar weight, age, delivery date, and when possible holding cage.
Blinding	In all in vivo assessment of survival and neurofunction, investigators who performed surgery and/or determined the outcome was blinded to the treatment (sulfide preconditioning, AAV-mediated SQOR knockdown or overexpression, sulfide scavengers), gender, or genotype of the animals. In all ex vivo and in vitro experiments in which levels of metabolites, mitochondrial function, viable or dead cell numbers, proteins, and mRNA were determined in tissue extracts or brain sections obtained from animals or in cultured cells or cell lysates, investigators who conducted measurements and data analysis were blinded to the treatment, gender, or genotype of the animals.

Reporting for specific materials, systems and methods

We require information from authors about some types of materials, experimental systems and methods used in many studies. Here, indicate whether each material, system or method listed is relevant to your study. If you are not sure if a list item applies to your research, read the appropriate section before selecting a response.

Materials & experimental systems

n/a	Involved in the study	
☐	☒	Antibodies
☐	☒	Eukaryotic cell lines
☒	☐	Palaeontology and archaeology
☐	☒	Animals and other organisms
☒	☐	Human research participants
☒	☐	Clinical data
☒	☐	Dual use research of concern

Methods

n/a	Involved in the study	
☒	☐	ChIP-seq
☒	☐	Flow cytometry
☒	☐	MRI-based neuroimaging

Antibodies

Antibodies used	GAPDH (Cell Signaling, 5174, lot#4, 1:5,000), GAPDH (Santa Cruz Biotechnology, Santa Cruz, clone: sc-25778, 1:5,000), vinculin (Cell Signaling, 4650, lot 4, 1:2,000), SQOR (Proteintech, 17256-1-AP, lot 00008637, 1:1,000), SQOR (house-made monoclonal antibody, 1:5,000), TST (GeneTex, GTX114858, lot 40233), ETHE1 (GTX115707, lot 40296), SUOX (Abnova, H00006821-D01, lot 11285), CBS (Cell Signaling, 14782, lot 1, 1:5,000), CSE (Proteintech, 12217-1-AP, lot 00022861, 1:1,000), 3MST (Sigma-Aldrich, HPA001240, A01741, 1:1,000), SDHA (Abcam, ab14715, 1:5,000), VEGF (Thermo Scientific, 710151, lot QG2060805, 1:1,000), GLUT-1 (Abcam, ab115730, 1:1,000), LDHA (Cell Signaling, 2012, lot# 2, 1:1,000), Anti-rabbit IgG, HRP-linked Antibody (Cell Signaling, 7074, 1:5,000 for Fig. 3d and Fig. S6a. 1:1:0,000 for the others), GFP (AvesLabs, GFP-1010, 1:1,000), Alexa Fluor® 488 conjugated anti-chicken IgY antibody (LifeTechnology, A-11039, 1:300)
Validation	<Immunoblotting><br>All primary antibodies used for immunoblotting were rabbit origin.<br><br>2240 publications have references monoclonal antibody against GAPDH (Cell Signaling , 5174, ~37 kDa). https://www.cellsignal.com/products/primary-antibodies/gapdh-d16h11-xp-rabbit-mab/5174

119 publications have references polyclonal antibody against GAPDH (Santa Cruz Biotechnology, Santa Cruz, clone: sc-25778, ~37 kDa). https://www.citeab.com/antibodies/795238-sc-25778-gapdh-antibody-fl-335

80 publications have references polyclonal antibody against vinculin (Cell Signaling, 4650, ~124 kDa). https://www.cellsignal.com/products/primary-antibodies/vinculin-antibody/4650

15 publications have references polyclonal antibody against SQOR (Proteintech, 17256-1-AP, ~50 kDa). Proteintech website: https://www.ptglab.com/products/SQRDL-Antibody-17256-1-AP.htm#tested-applications. This antibody was used for Fig. 1n and o, Fig. 2b and e, Fig. 4g, Fig. 5a, Fig. S1d and f, Fig. S2a, Fig. 3Sa, Fig. S7e, Fig. S8a, Fig. S11h. Although we did not examine interspecies (mouse, rat, and 13-LGS) cross-reactivity of this antibody in Fig. 4g and Fig. S7c, we confirmed enzymatic activity of tissue SQOR shows the same trend as the result of WB in Fig. 4h and Fig. S7d.

SQOR (house-made, monoclonal antibody) was used for Fig. 3d and Fig. S6a. We confirmed that this antibody reacts to only mitochondrial fraction but not cytosolic fraction of the protein extract from wild-type mice. This antibody also reacts to only cytosolic fraction but not mitochondrial fraction of the brain protein extract from Δ14/Δ14 mice. These observations validate this antibody because SQOR locates only in mitochondria or cytosol in wild-type or Δ14/Δ14 mice, respectively.

4 publications have references monoclonal antibody against CBS (Cell Signaling, 14782, ~61 kDa). https://www.cellsignal.com/products/primary-antibodies/cbs-d8f2p-rabbit-mab/14782

76 publications have references polyclonal antibody against CSE (Proteintech, 12217-1-AP, ~42 kDa). https://www.thermofisher.com/antibody/product/Gamma-cystathionase-Antibody-Polyclonal/12217-1-AP

11 publications have references polyclonal antibody against 3MST (Sigma-Aldrich, HPA001240 ~38 kDa). https://www.atlasantibodies.com/products/antibodies/primary-antibodies/triple-a-polyclonals/mpst-antibody-hpa001240/

294 publications have references monoclonal antibody against SDHA (Abcam, ab14715, ~70 kDa). https://www.abcam.com/sdha-antibody-2e3gc12fb2ae2-ab14715.html

A publication has references polyclonal antibody against VEGF (Thermo Scientific, 710151, ~40 kDa). https://www.thermofisher.com/antibody/product/VEGF-Antibody-clone-1HCLC-Recombinant-Polyclonal/710151

95 publications have references monoclonal antibody against GLUT-1 (Abcam, ab115730, ~54kDa). https://www.abcam.com/glucose-transporter-glut1-antibody-epr3915-ab115730.html?productWallTab=ShowAll

68 publications have references polyclonal antibody against LDHA (Cell Signaling, 2012, ~37 kDa). https://www.cellsignal.com/products/primary-antibodies/ldha-antibody/2012

5984 publications have references goat anti-rabbit IgG, HRP-linked Antibody (Cell Signaling, 7074). https://www.cellsignal.com/products/secondary-antibodies/anti-rabbit-igg-hrp-linked-antibody/7074

<Immunofluorescence>
We used the following primary and the 2nd antibodies for immunofluorescence to detected GFP in Fig. 2k and l.

49 publications have references anti-GFP antibody (AvesLabs, GFP-1010). https://www.aveslabs.com/products/green-fluorescent-protein-gfp-antibody

214 publications have references Alexa Fluor® 488 conjugated anti-chicken IgY antibody (LifeTechnology, A-11039). https://www.thermofisher.com/antibody/product/Goat-anti-Chicken-IgY-H-L-Secondary-Antibody-Polyclonal/A-11039

Eukaryotic cell lines

Policy information about cell lines

Cell line source(s)	SH-SY5Y (ATCC, CRL-2266, batch# 63724189)
Authentication	We purchased SH-SY5Y cells from ATCC. This cell line was authenticated by ATCC using ampule passage number, population doubling level (PDL), total cells/ampule, post-freeze viability, growth properties, morphology, test for mycoplasma contamination, species determination: COI assay (interspecies), species determination: STR analysis (intraspecies), sterility test (BacT/ALERT 3D), and human pathogenic virus testing (PCR-based assay for HIV, HepB, HPV, EBV, and CMV).
Mycoplasma contamination	SH-SY5Y cells were tested negative for mycoplasma by ATCC.
Commonly misidentified lines (See ICLAC register)	No commonly misidentified cell lines were used in this study.

Animals and other organisms

Policy information about studies involving animals; ARRIVE guidelines recommended for reporting animal research

Laboratory animals	Male C57BL6J mice age between 8 and 12 weeks old (Jackson Laboratory). Male and female CD1 mice age between 8 and 12 weeks old (Charles River laboratories). Male and female Sprague-Dawley rats age between 8 and 16 weeks old (Charles River Laboratories). Male and female 13 lined ground squirrels between 2 and 8 months old.

Wild animals	The study did not involve wild animals.
Field-collected samples	The study did not involve the samples collected in the field.
Ethics oversight	All animal protocols were approved by the Massachusetts General Hospital Institutional Animal Care and Use Committee. Experimental procedures related to generation and initial characterization of Sqor dN/dN mice conformed to the Regulations for Animal Experiments and Related Activities at Tohoku University, were reviewed by the Institutional Laboratory Animal Care and Use Committee of Tohoku University, and were approved by the President of Tohoku University. Mice were housed under 12h light/12h dark cycle with room temperature and humidity at 21-23 degree Celsius and 40-60%, respectively.

Note that full information on the approval of the study protocol must also be provided in the manuscript.

This checklist template is licensed under a Creative Commons Attribution 4.0 International License, which permits use, sharing, adaptation, distribution and reproduction in any medium or format, as long as you give appropriate credit to the original author(s) and the source, provide a link to the Creative Commons license, and indicate if changes were made. The images or other third party material in this article are included in the article's Creative Commons license, unless indicated otherwise in a credit line to the material. If material is not included in the article's Creative Commons license and your intended use is not permitted by statutory regulation or exceeds the permitted use, you will need to obtain permission directly from the copyright holder. To view a copy of this license, visit http://creativecommons.org/licenses/by/4.0/
